# Variation in Phenolic Chemistry in *Zostera marina* Seagrass along Environmental Gradients

**DOI:** 10.3390/plants10020334

**Published:** 2021-02-09

**Authors:** Cecilie Sævdal Dybsland, Trine Bekkby, Kjersti Hasle Enerstvedt, Olav M. Kvalheim, Eli Rinde, Monica Jordheim

**Affiliations:** 1 Department of Chemistry, University of Bergen, Allégt. 41, N-5007 Bergen, Norway; Cecilie.Dybsland@uib.no (C.S.D.); kjersti.enerstvedt@uib.no (K.H.E.); olav.kvalheim@uib.no (O.M.K.); 2 Section for Marine Biology, Norwegian Institute for Water Research (NIVA), Gaustadalléen 21, N-0349 Oslo, Norway; trine.bekkby@niva.no (T.B.); eli.rinde@niva.no (E.R.)

**Keywords:** *Zostera marina*, sulfated flavonoids, rosmarinic acid, zosteric acid, wave exposure, quantification, multivariate analysis, chemical ecology, seagrass monitoring

## Abstract

Chemical ecology has been suggested as a less time-consuming and more cost-efficient monitoring tool of seagrass ecosystems than traditional methods. Phenolic chemistry in *Zostera marina* samples was analyzed against latitude, sea depth, sample position within a seagrass meadow (periphery or center) and wave exposure. Multivariate data analysis showed that rosmarinic acid correlated moderately positively with depth, while the flavonoids had an overall strong negative correlation with increasing depth—possibly reflecting lack of stress-induced conditions with increasing depth, rather than a different response to light conditions. At a molecular level, the flavonoids were separated into two groups; one group is well described by the variables of depth and wave exposure, and the other group that was not well described by these variables—the latter may reflect biosynthetic dependencies or other unrevealed factors. A higher flavonoid/rosmarinic acid ratio was seen in the periphery of a seagrass meadow, while the contrary ratio was seen in the center. This may reflect higher plant stress in the periphery of a meadow, and the flavonoid/rosmarinic acid ratio may provide a possible molecular index of seagrass ecosystem health. Further studies are needed before the full potential of using variation in phenolic chemistry as a seagrass ecosystem monitoring tool is established.

## 1. Introduction

Seagrasses are rooted vascular flowering plants (marine angiosperms) originating from land, often forming meadows in relatively shallow areas along the coast. Despite only covering less than 0.2% of the global sea surface area [1,2], they play an important role in the coastal ecosystem, as they often are the only habitat building species on shallow soft sediment. Seagrasses consist of about 60 different species with more than one thousand associated species of flora and fauna utilizing the seagrass habitat [3]. Seagrasses are in decline globally, and the rate of loss of area has increased from less than 1% per year before 1940 to 7% per year since 1990, resulting in a total loss of 29% of the known areal extent since the first seagrass recording in 1879 [4,5]. Seagrasses are threatened by many factors, such as physical modification, nutrient overload, sedimentation, the introduction of non-native species, overexploitation and climate change. The two major causes of the observed seagrass loss are, however, related to coastal development and degraded water quality [4]. Monitoring seagrass health is important to be able to detect and mitigate any severe impact of these direct and indirect human impacts on these valuable ecosystems and seagrass health indices may serve as indicators of coastal health in general. Seagrass monitoring has traditionally been based on long-term observations of changes in seagrass parameters, such as species composition, percent cover, biomass and plant morphometry that are identifiable in the field ([6,7] and refs therein). To date, molecular methods as tools for assessing plant health have been little examined. Seagrass phenolics have a high potential in this respect, as they are reckoned to play an important role in plant protection and appear to be affected by several biotic and abiotic pressures [8,9,10,11,12,13,14,15,16,17,18,19].

Phenolic chemistry has been tested as a putative descriptor of seagrass health or ecological status [7,17,20,21,22]. Migliore et al. examined *Posidonia oceanica* and related the spatial heterogeneity of environmental pressures to variations in the total phenolic content, which was further linked to differences in metabolic/physiological pathways as an adaptation to stress [17]. The total phenolic content was also found to be inversely related to the density of seagrass plants within meadows and linked to less stressful conditions and a better health state in meadows with high plant density than within meadows with low plant density [17,20]. Despite some research on seagrass phenolics as possible biochemical markers in seagrass monitoring, examinations at a differentiated molecular level are relatively little explored. Studies of the phenolic content of seagrasses at a molecular level often focus on simple phenolic acids [12,15,16,21,23] and not the more structurally advanced and diverse group of flavonoids. Böttner et al. (2020) recently reported that individual flavonoid glucosides have distinct cellular and subcellular locations and promote duckweed (*Spirodela polyrhiza*, aquatic plant) fitness under different abiotic stresses [24]. In general, the ecological roles of flavonoids are far from understood, partially due to their structural diversity, different accumulation patterns and varying abundance, all of which affect their biological activity [24,25,26].

Among the seagrasses, *Zostera marina* L. (eelgrass) is the most widely distributed species in the northern hemisphere, reflecting a broad tolerance of temperature, salinity and photoperiod conditions [27]. In Norway, the species is commonly found as large meadows with high plant density in the southern parts of Norway. The species is also found further north all the way to the northermost region (70° N), but then often as smaller meadows or more scattered occurrences. The existence of sulfated flavonoids in *Zostera* spp. is well-documented [9,28,29,30], and the appearance of these compounds has been suggested to be related to high concentration of sulfate ion in seawater [30]. The physiological role of the sulfated flavonoids in plants and the factors that regulate flavonoid sulfate accumulation is unclear. However, some evidence implies physiological protection of the seagrass [30,31,32,33]. As for the phenolic acid content of *Z. marina*, zosteric acid (ZA), a sulfated phenolic acid only seen in *Zostera* species [11], has been found to prevent the settlement of marine bacteria, algae, barnacles and tubeworms [34]. Rosmarinic acid (RA), another phenolic acid, widely known for its biological activities, including antioxidant and antifouling action [35,36], has been identified as one of the major compounds in *Z. marina* [23,29,37].

The main aim of this study was to analyze and assess any systematic variation in phenolic acids (ZA, RA) and flavonoids in *Zostera marina*, at a molecular level, along environmental gradients (latitude, depth as a proxy for the reduction in light conditions, wave exposure, and meadow positioning). The secondary aim was to identify any specific biochemical markers or molecular metrics that can be used in the context of seagrass health and monitoring.

## 2. Results

### 2.1. Phenolic Profiles

**Figure 1:**
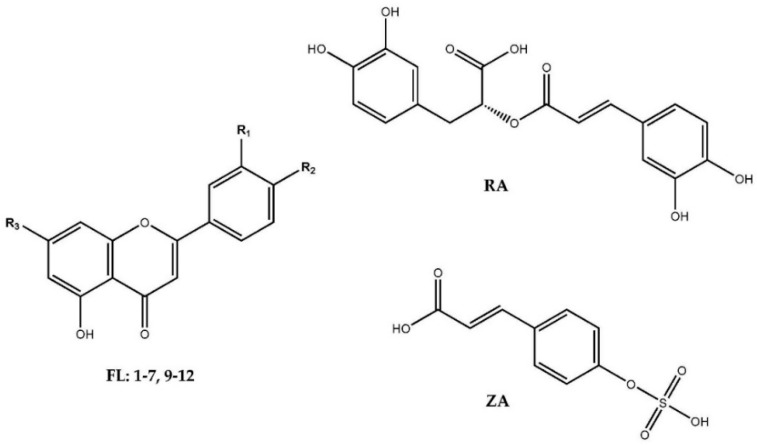
Structures of the flavonoids and phenolic acids found in the examined *Z. marina* samples collected. FL: **1** = luteolin 7,3′-disulfate (R_1_ = OH, R_2_ = R_3_ = SO_3_OH), **2** = diosmetin 7,3′-disulfate (R_1_ = OCH_3_, R_2_ = R_3_ = SO_3_OH), **3** = luteolin 7-glucoside (R_1_ = R_2_ = OH, R_3_ = glucoside), **4** = luteolin 7-sulfate (R_1_ = R_2_ = OH, R_3_ = SO_3_OH), **5** = apigenin 7-glucoside (R_1_ = OH, R_2_ = H, R_3_ = C_6_H_12_O_6_), **6** = luteolin 7-(6″-malonyl)glucoside (R_1_ = R_2_ = OH, R_3_ = 6″-malonyl-glucoside), **7** = luteolin 3′-sulfate (R_1_ = R_3_= OH, R_2_ = SO_3_OH), (**8** = apigenin 7-sulfate (R_1_ = OH, R_2_ = H, R_3_ = SO_3_OH)), **9** = chrysoeriol 7-sulfate (R_1_ = OH, R_2_ = OCH_3_, R_3_ = SO_3_OH), **10** = diosmetin 7-sulfate (R_1_ = OCH_3_, R_2_ = OH, R_3_ = SO_3_OH), **11** = apigenin 7-(6″-malonyl)glucoside (R_1_ = OH, R_2_ = H, R_3_ = 6″-malonyl-glucoside), **12** = luteolin (R_1_ = R_2_ = R_3_ = OH), RA = rosmarinic acid, ZA = zosteric acid.

The HPLC-DAD and LR-LCMS analyses of *Z. marina* extracts revealed the same polyphenolic content as previously reported; eleven flavones (**1–7**, **9**–**12**) and two phenolic acids (ZA, RA) [28,29,38] (Figure 1, Table 1 and Table 2).

The sulfated flavonoids **1**, **2**, **4**, **10** and luteolin (**12**) were found in all the examined samples, covering the total range of the latitudinal gradient (see Section 4.1), as did rosmarinic acid (RA) and zosteri acid (ZA). Chrysoeriol 7-sulfate (**9**) was also present in most samples, with Munkefjorden (M) and Rafsbotn (N) as the only exceptions. Luteolin 7-glucoside (**3**) was found in three localities along the latitudinal gradient; furthest south (A), north-west (G_1_) and north (N). Compound **3** has previously been found in samples from Hordaland (B) as well [29]. Luteolin 7-(6″-malonyl)glucoside (**6**) and luteolin 3′-sulfate (**7**) were also found along a wide range of the latitudinal gradient.

**Figure 2:**
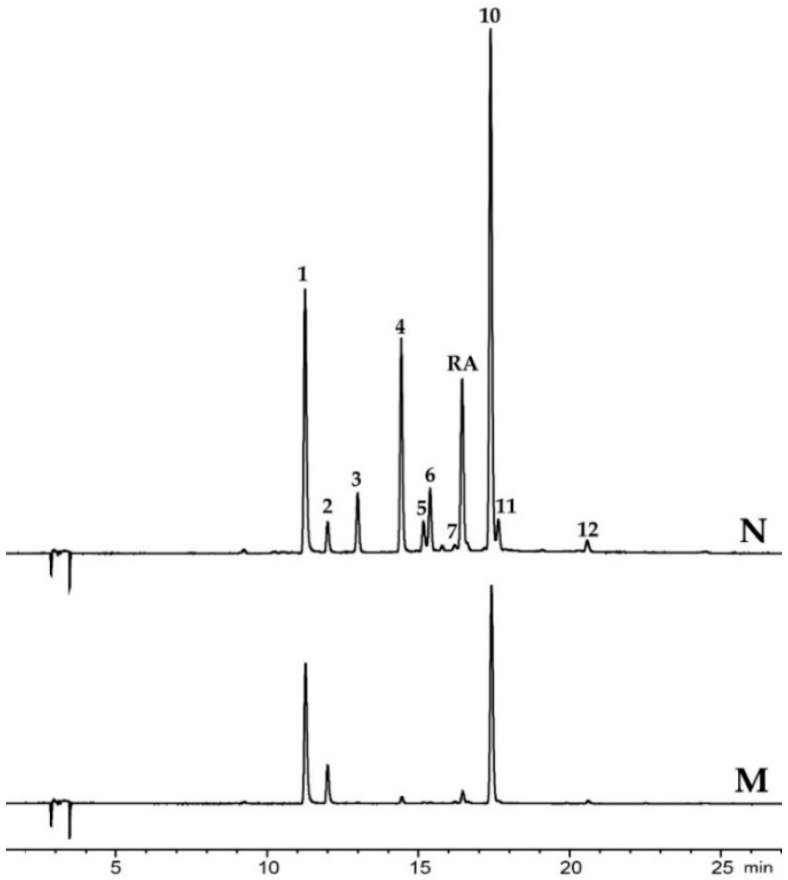
HPLC-DAD chromatograms recorded at 360 ± 20 nm for the two northernmost *Zostera marina* samples (~70° N, 27° E; M; Munkefjorden, N; Rafsbotn).

The two samples from the northernmost region (~70° N, 27° E; M, N) (Figure 2), differed greatly in the occurrence of individual flavonoids (Table 1 and Table 2).The two apigenin derivatives, **5** and **11**, were only observed in one of these samples (N). Apigenin 7-glucoside (**5**) and apigenin 7-sulfate (**8**) have previously been found in small amounts in *Z. marina* and in *Z. noltii* from Hordaland, while apigenin 7-(6″-malonyl)glucoside (**11**) has only been found in *Z. noltii* previously [29]. Apigenin 7-sulfate (**8**) was not found in any of the samples in the current study.

An average flavonoid amount of 6.24 ± 0.21 mg luteolin Eq./g dry weight (DW) was observed for the 24 *Z. marina* samples (Table 2). The highest amounts were found in Stormalen (G_1_) (10.20 ± 0.35 mg luteolin Eq./g DW) and Larvik (A) (10.23 ± 0.77 mg luteolin Eq./g DW). The lowest amounts were found in one of the highest latitude samples, Munkefjorden (M) (2.35 ± 0.41 mg luteolin Eq./g DW). The two seagrass samples from Finnmark region (M, N) had different qualitative profiles, and the quantitative amounts were higher in Rafsbotn (N) compared to Munkefjorden (M), with one exception. The amount of diosmetin 7,3′-disulfate (**2**) in Munkefjorden (M) (0.11 ± 0.04 mg luteolin Eq./g DW) exceeded the corresponding amount found in Rafsbotn (N) (0.06 ± 0.01 mg luteolin Eq./g DW), accounting for 4.75% and 0.8% of total amounts, respectively. The apigenin derivatives (**5**, **11**) found in Rafsbotn (N) accounted for 2.2% of the total flavonoid content in this sample.

A correlation between luteolin 7-glucoside (**3**) and luteolin 7-sulfate (**4**) was observed. Luteolin 7-glucoside (**3**) was exclusively present in samples A, G_1_ and N, with high amounts of luteolin 7-sulfate (**4**). The observation was independent of where in Norway the samples were collected and is most likely related to the enzymatic process for the substitution of the 7-position on the ring.

For the phenolic acids, the western Møre and Romsdal sample **I** showed the highest content of rosmarinic acid (RA) (4.53 ± 0.31 mg luteolin Eq./g DW), approximately 29.5% of the total phenolic content, with zosteric acid (ZA) making 4.4% of the total content. The other nineteen samples in the Møre and Romsdal region (C-L) had lower RA content relative to the total phenolic content; 2-17%—while the ZA content was ranging from 0.1% to 4%. In the samples A (59° N, 10° E), B (60° N, 5° E) and G_2_ (63° N, 6.5° E) the highest amounts of ZA were found—with G_2_ showing the highest relative content with 5.6% ZA of total phenolics.

### 2.2. Relative Amount of Sulfated Flavonoids within Regions

**Figure 3:**
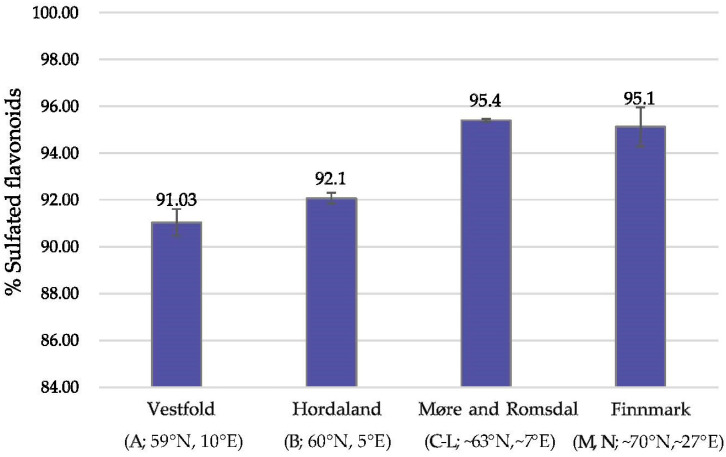
Relative sulfated flavonoid content (%) (**1**, **2**, **4**, **7**, **9**, **10**, Figure 1) found in *Zostera marina* leaves collected from localities in different regions (Vestfold, Hordaland, Møre and Romsdal, Finnmark, see Section 4.1) in Norway.

A comparison of the relative amount of sulfated flavonoids (**1**, **2**, **4**, **7**, **9**, **10**) in the *Z. marina* leaves between regions was made based on the average values within sampled regions (Figure 3 and Section 4.1). The northernmost regions, Finnmark (M, N; ~70° N, ~27° E) and Møre and Romsdal (C-L; ~63° N, ~7° E), had the relatively highest sulfated flavonoid content compared to the two regions further south, Hordaland (B; 60° N, 5° E) and Vestfold (A; 59° N, 10° E). There were no significant differences between the average sulfated flavonoid amounts found in Finnmark (M, N) compared to Møre and Romsdal (C-L). However, between the colder west coast of Hordaland (B) and the south situated Vestfold (A), a small significant difference was observed (*p* < 0.05).

### 2.3. Phenolic Content—Seagrass Meadow Positioning

**Figure 4:**
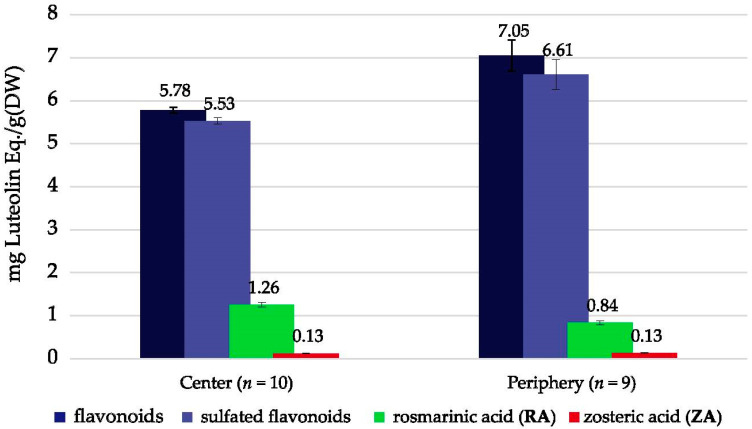
Average of total flavonoids (TF), sulfated flavonoids (TSF), rosmarinic acid (RA) and zosteric acid (ZA) in selected *Zostera marina* samples (*n* = 19) with different positions in seagrass meadows (center samples, *n* = 10 and periphery samples, *n* = 9).

In order to examine the phenolic content in samples with different positions in meadows, center and periphery species were analyzed from selected meadows (*n* = 19). An average concentration of total flavonoids (TF), sulfated flavonoids (TSF), rosmarinic acid (RA) and zosteric acid (ZA) was made for the two different positions (Figure 4). The flavonoids showed an average of 17% higher concentrations in the samples collected from the periphery (*n* = 9) than in the samples collected from the center (*n* = 10). Interestingly, and in contrast to the flavonoids, the average rosmarinic acid (RA) concentration was found to be 30% lower in the periphery samples compared to the center samples. For the small amounts of zosteric acid (ZA), no difference was observed between the two meadow positions.

### 2.4. Phenolic Content—Variations between Wave Exposure Classes

**Figure 5:**
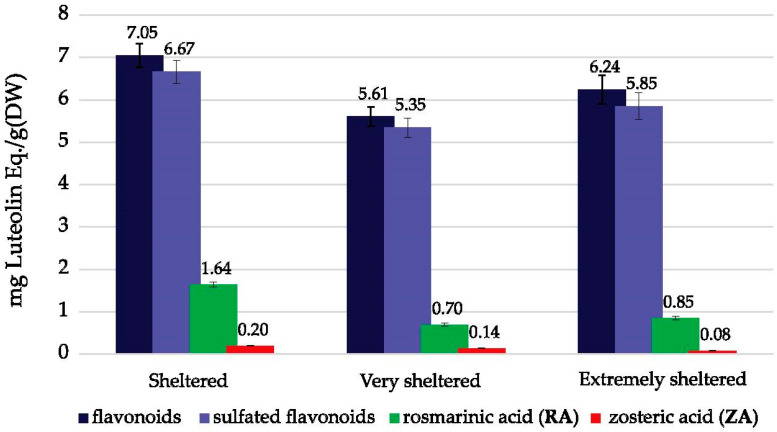
Average values of total flavonoids (TF), total sulfated flavonoids (TSF), rosmarinic acid (RA) and zosteric acid (ZA) content for the 24 *Zostera marina* samples in the dataset (A-N, see Section 4.1).

The analyzed *Z. marina* leaves were sampled within three classes of wave exposure; “extremely sheltered”, “very sheltered,” and “sheltered” see Section 4.1 and Section 4.2). The average values of total flavonoids (TF), sulfated flavonoids (TSF), rosmarinic acid (RA) and zosteric acid (ZA) content of the sampled leaves within each category were calculated (Figure 5).

Samples collected from the “sheltered” areas, representing the highest wave exposure values in this dataset, showed higher amounts of flavonoids than the “very sheltered” and “extremely sheltered” categories, 20% and 12%, respectively. For rosmarinic acid (RA), the “sheltered” samples had as much as 57% higher RA content than the “very sheltered” samples. Although the analytical amounts of zosteric acid (ZA) are low, a steady increase with respect to wave exposure was observed.

### 2.5. Variations in Phenolic Content Explained by Depth and Wave Exposure

**Figure 6:**
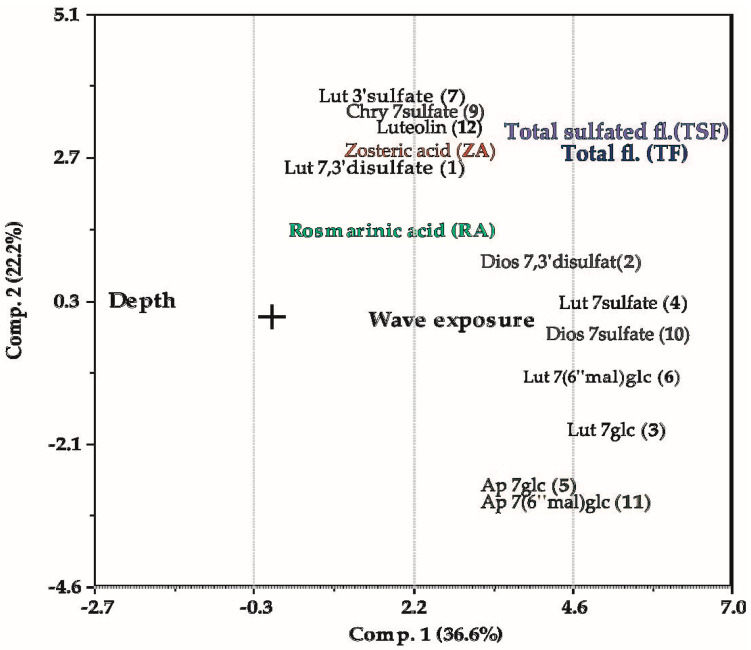
PCA loadings (PC1 vs. PC2), showing the correlations between variables depth, wave exposure, flavonoids (**1**–**7**, **9**–**12**, TF and TSF) and phenolic acids (RA, ZA) in the current *Zostera marina* dataset (see Figure 1 for structures).

**Figure 7:**
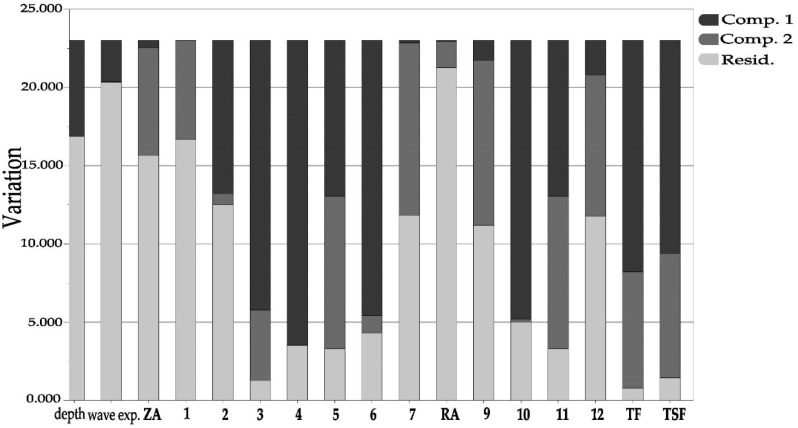
Variation plot for the extracted components 1 (PC1) and 2 (PC2) of the variables in the *Zostera marina* dataset. See Figure 1 for structures.

The PCA explained 58.8% of the total variation (PC1; 36.6% and PC2; 22.2%). The cosine of the angle between the loadings of pairs of variables represents their correlation in the PCA-plot (Figure 6). A negative sign means that the variables are negatively correlated in the model. As the variables were standardized, a position close to zero implies that this particular variable does not correlate with the variation that component 1 and 2 is reflecting (Figure 6) [39]. Figure 7 shows the quantitative contribution of individual variables to PC1 and PC2, as well as the variation in each variable not explained by the two-component PCA model.

The multivariate analysis indicates moderate negative correlations between depth and the total amount of flavonoids (TF) and sulfated flavonoids (TSF) (Figure 6). As shown by the low residual variance (Figure 7), the TF and TFS variations are well described by components 1 and 2. Interestingly, there are differences with respect to how well the PCA plot describes the variance of the individual flavonoids. The flavonoids luteolin-7,3′-disulfate (**1**), luteolin 3′-sulfate (**7**), chrysoeriol 7-sulfate (**9**) and luteolin (**12**), seem not to be well-described by depth, while the flavonoids; diosmetin 7,3′-disulfate (**2**), luteolin 7-glucoside (**3**), luteolin 7-sulfate (**4**), luteolin 7-(6″-malonyl)glucoside (**6**) and diosmetin 7-sulfate (**10**), are all strongly negatively correlated with depth. The two apigenin derivatives (**5**, **11**), found in the north (M), have almost identical positions in the plot—and they are 100% correlated with each other. They (**5**, **11**) correlate weakly negatively with the total flavonoid amounts (TF, TSF) and moderately negatively with depth.

Both TF and TSF correlate moderately positively with wave exposure. The exceptions are again the flavonoids; **1**, **7**, **9** and **12**, showing only weak correlations to wave exposure—repeating their response pattern relative to flavonoids **2**–**3**, **6** and **10**—as seen for the depth correlation. The flavonoids **1**, **7**, **9** and **12** are also the ones among the flavonoids having their variances less described by the “multiple variable” PCA plot (Figure 7). However, PCA analysis for individual flavonoids with depth only and with wave exposure only resulted in the same trends for **1**, **7**, **9** and **12**, both with respect to correlations coefficients (Figure 6), and the lack of good variance description for these flavonoids (Figure 7).

The phenolic acids (ZA, RA) correlate weakly to wave exposure and depth in the “multiple variable” PCA-loading plot (Figure 6). However, Figure 7 shows that the variance seen in RA and ZA is not well described by components 1 and 2. Figure 5 shows a wave exposure dependent trend also for these two phenolic acids. The PCA partial correlation was tested for the phenolic acids, including only the variables depth and wave exposure, describing 71% of the variance (Table 3). The PCA analysis revealed a strong correlation for both acids with wave exposure and a moderate positive correlation to depth for RA, and a moderate negative correlation to depth for ZA. The depth and wave exposure are not correlated.

## 3. Discussion

The current study supplement our earlier studies of phenolics in Norwegian *Zostera marina* meadows [28,29,38] by including a larger latitudinal gradient (from just 60° N to covering 58–70° N). This gave us new insight into the phenolic chemistry of *Z. marina* in the northern areas, which has never been studied before. In addition, and importantly, our new dataset also contained parameters as depth (as a proxy for the reduction in light conditions), wave exposure and meadow positioning—improving chemoecological examinations.

Based on the qualitative phenolic profiles, the *Z. marina* samples examined in this study seems to reflect only one chemotype [30]. However, the northernmost *Z. marina* sample (N; 70° N, 23.5° E) had the highest diversity of flavonoids in the dataset—despite the environmental limitations *Z. marina* probably meet in this part of Norway (Table 1 and Table 2) [41]. This location also had relatively high total flavonoid amounts (7.57 ± 0.58 mg luteolin Eq./g DW), and one of the highest productions of diosmetin 7-sulfate (**10**) (3.58 ± 0.35 mg luteolin Eq./g DW, 47.3%). Only comparable to the amounts found in the southernmost sample (A; 59° N, 10° E: 3.92 ± 0.42 mg luteolin Eq./g DW, 38.3%). Little is known about flavonoid plant functionality at a molecular level. Recently, Papazian et al. examined the surface chemical defense of *Z. marina* against microbial fouling, where they differentiate between surface extract and C18 extract [19]. They found that the most abundant phenolic detected on the surface was diosmetin 7-sulfate (**10**), with 10-fold higher concentrations than the C18 extract. The next most abundant phenolic in the surface extract (absent in the C18) was ZA, followed by lower concentrations of caffeic acid, ferulic acid, *p*-coumaric acid, and the sulfated flavonoids luteolin 7-sulfate (**4**) and apigenin 7-sulfate (**8**). Notably, RA had only trace levels in surface extracts (<0.60 ng.mL/1). Whether the higher diosmetin 7-sulfate (**10**) amounts seen in our study could be linked to chemical defense or not warrants further examination. The northernmost *Z. marina* sample (N; 70° N, 23.5° E) also contained 2.2% apigenin derivatives (**5**, **11**)—not seen in the rest of the samples. However, apigenin 7-sulfate (**8**) and apigenin 7-(6″-malonyl)glucoside (**11**) were found in spring samples of *Z. marina* in our previous studies (1–4%) (Hordaland, B; 60° N, 5° E), while apigenin 7-glucoside (**5**) was not found [29]. The samples in the current study were collected in late summer/fall. It was suggested that decreasing temperature could cause a shift in secondary metabolite profiles, hence a change in the qualitative profile of flavonoids—possibly affecting the presence of apigenin in the northernmost samples [14,42,43]. However, the N neighboring northern locality, Munkefjorden (M; 69.7° N, 29.5° E), is lacking apigenin derivatives—in addition to the absence of high flavonoid diversity (Figure 2). The samples from M were collected at lower depth and less wave exposure than in N. The fjord is also exposed to erosion, resulting in increased amounts of particles in the water—possibly limiting the light availability and hence the biosynthesis.

Our previous study on *Z. marina* from two different localities in Hordaland, showed that the sample from the southernmost area contained the relatively lowest amounts of sulfated flavonoids [29]. In the same study, regional differences in amounts of sulfated flavonoids in *Z. noltii* were also seen. The two samples from Vestfold (59° N, 10° E) had significantly lower relative amounts of sulfated flavonoids than the populations on the colder west coast. These observations are in agreement with the observations in the present study (Figure 3, see also Section 4.1). Several studies have shown that low water temperatures result in an increase in flavonoid production [14,15,43]. It seems, though, to be an increase in the occurrence of the sulfated flavonoids related to the longitude and colder climatic or harsher conditions, possibly reflecting the indicated physiological protective role of sulfated flavonoids in the seagrass [30,31,32,33]. However, in *Zostera* spp. the production of the major flavonoid group—the sulfated flavonoids, depends on the total flavonoid biosynthesis. Hence, further studies are needed since this observation is not independent of the total flavonoid production in the plant.

For 19 of the 24 samples in the dataset, the position of the sample in the seagrass meadow was registered. Samples collected in the center (*n* = 9) had on average a significantly lower flavonoid amount (TF/TSF) compared to the samples at the periphery, possibly reflecting differences in light exposure, available nutrients or external stress. Interestingly, and in contrast to the flavonoids, the average rosmarinic acid (RA) concentration was found to be 30% lower in the periphery samples compared to the center samples. For the small amounts of zosteric acid (ZA), no difference was observed between the two meadow positions. The PCA modeling indicates that TF/TSF correlates moderately positively to wave exposure (Figure 6), while RA and ZA are strongly positively correlated (Table 3). This is evident from Figure 5. For rosmarinic acid (RA), the “sheltered” (most wave-exposed) samples had as much as 57% higher RA content compared to the “very sheltered” (more wave protected) samples. In comparison, the flavonoids showed a 20% increase in the most wave-exposed samples. Although the analytical amounts of zosteric acid (ZA) were low, a steady increase with respect to wave exposure was observed. Thus, explaining the higher concentration of RA in center samples compared to periphery samples, opposite to what is seen for the flavonoids, appears to be difficult. Do the samples in the center experience decreased nutrient accessibility, higher competition and lower wave exposure compared to the periphery samples? The center samples most likely experience lower physical stress and possibly also lower light exposure due to higher biomass density. Flavonoids are suggested to be relatively poor UV-B-absorbers compared to other phenylpropanoids, such as hydroxycinnamic acids [44,45]. However, the ratio of flavonoids to hydroxycinnamic acids strongly increases upon exposure to UV-B or strong sunlight, and, according to the authors, these observations suggest that UV-B screening is not the sole function for flavonoids. However, the main role of the flavonoids seems to be to reduce oxidative stress since the flavonoid biosynthesis is upregulated by a plethora of abiotic and biotic stresses that all lead to the generation of reactive oxygen species (ROS) [24,46,47,48]. This indicates that the stress in the periphery of a meadow is higher than in the center. Seagrass meadows are ecologically valuable habitats, and pair-wise interactions have traditionally been used to study the implications of secondary metabolites in these systems. Phenolic amounts have been reported to vary within a meadow, possibly reflecting the heterogeneity of environmental pressures [17,20]. However, systematic differences in the presence of flavonoids and phenolic acids within a seagrass meadow have not been reported before, as far as we know. Whether the flavonoid/RA ratio at the periphery of a seagrass meadow could be used as a biochemical marker or molecular tool to assess the health of the meadows needs further exploration.

The PCA (Figure 6 and Figure 7, Table 3) indicates a moderate positive correlation with depth for RA and a moderate negative correlation for ZA. For the total flavonoids, we observed an overall strong negative correlation with increasing depth. The different response to depth seen for RA and total flavonoids may be interpreted as lack of stress-induced conditions with increasing depth, rather than a different response to light conditions. At the molecular level, a differentiation was observed within the flavonoids: the variation in luteolin-7,3′-disulfate (**1**), luteolin 3′-sulfate (**7**), chrysoeriol 7-sulfate (**9**) and luteolin (**12**), was not well described by depth nor wave exposure, while the flavonoids; diosmetin 7,3′-disulfate (**2**), luteolin 7-glucoside (**3**), luteolin 7-sulfate (**4**), luteolin 7-(6″-malonyl)glucoside (**6**) and diosmetin 7-sulfate (**10**), were all strongly negatively correlated with depth and moderately positively correlated with wave exposure. The reasons for this are unclear. However, it may reflect their biosynthetic dependencies or independencies to each other. It may also reflect an unrevealed function of these flavonoids within the seagrass—not evaluated in this study. Further studies are needed to understand the additional roles of these secondary metabolites and their ecological interaction strategies in *Z. marina.*

## 4. Materials and Methods

### 4.1. Study Sites, Plant Collection and Explanatory Variables

**Figure 8:**
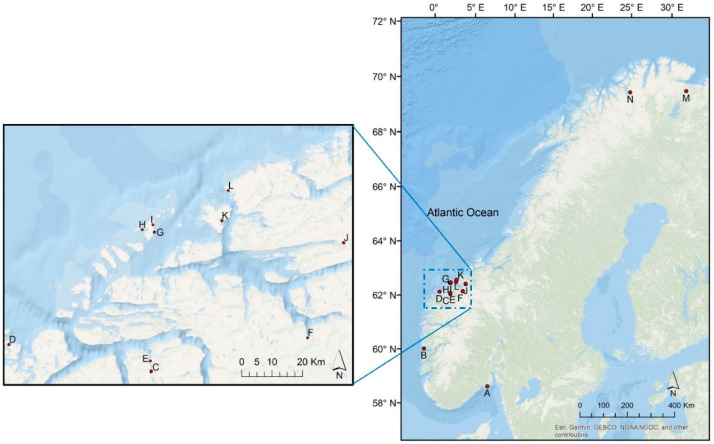
Sample localities (A–N) (Table 4) of collected *Zostera marina* samples along the Norwegian coast.

The plant material of *Z. marina* was collected from fourteen different locations in Norway (Table 4, Figure 8), covering ecoregions for Skagerrak to the Barents Sea, including Vestfold, (59° N, 10° E; A), Hordaland (60° N, 5° E; B), Møre and Romsdal (~63° N, 7° E; C-L) and Finnmark (~70° N, 27° E; M, N). The seagrass plants were collected in the period from late summer to autumn in 2017 and 2018 during the mapping of the *Z. marina* meadow distribution as in the National Program for Mapping of Biodiversity—Coast [49] and during fieldwork in the EU funded project MERCES [50]. The specimens were collected in meadows with moderate to high plant density to avoid potential impacts of variation in *Z. marina* density. Furthermore, the sampling was designed to cover the widest possible range of wave exposure (covering “sheltered”, “very sheltered” and “extremely sheltered” areas) (see Section 4.2). Sampling was done using a throw rake or by snorkeling, collecting 10–20 leaves. Immediately after sampling, the leaves were carefully cleaned in saltwater for particles and epiphytic algae and fauna. Back on land (after a maximum of a couple of hours), the leaves were rinsed in fresh water and air dried (away from sunlight) before being shipped for analyses. The site of the sampling was georeferenced using a GPS with approx. 2 m accuracy, and depth was recorded for each station using a handheld depth sensor. At the office, wave exposure values (modeled as continuous values, as described in, e.g., Bekkby et al. (2008)) were assigned to all samples [51]

### 4.2. The Wave Exposure Model

The wave exposure model (m^2^/s) was developed with a spatial resolution of 25 m, based on fetch (distance to nearest shore, island or coast), averaged wind speed and wind frequency (estimated as the amount of time that the wind comes from one of 16 direction). Data on wind speed and direction were delivered by the Norwegian Meteorological Institute and averaged over a 10 year period (i.e., 1995-2004), and therefore provides an estimate of the relative differences in wave exposure between sites, not the exact wave exposure at each station at the time of sampling. The model was developed by NIVA as a part of the National Program for Mapping of Biodiversity—Coast [49] and has been applied in several research projects in Norway [51,53,54,55,56,57,58], Sweden [59], Finland [60], the Danish region of the Skagerrak coast and the Russian, Latvian, Estonian, Lithuanian and German territories of the Baltic Sea [61].

### 4.3. Analytical Instrumentation

Analytical HPLC: The HPLC-DAD analyses were performed using an Agilent 1260 Infinity series quaternary pump system (Agilent Technologies, Santa Clara, CA, USA) with an Agilent 1200 series diode array detector (DAD). The analysis was performed using two solvents, (A) super distilled water with 0.5% TFA and (B) acetonitrile with 0.5% TFA. The initial conditions were 90% A and 10% B, followed by a linear gradient to 50% B. Aliquots of 20 μL were injected using an Agilent 1100 series autosampler, and the flow rate was 1 mL/min. UV-vis absorption spectra were recorded online during HPLC analysis with a wavelength range of 190–600 nm in the step of 2 nm. An Agilent Hypersil 5 μm ODS, 250 × 4.6 mm, column was used for flavonoid analysis.

Low-resolution LC-electrospray mass spectrometry (LR-LCMS) (ESI+/ESI−) was performed using an Agilent 1260 Infinity series system in combination with Agilent Technologies 6420A triple quadrupole mass spectrometry detector. The following instrumental conditions were applied: ionization mode: positive/negative, capillary voltage = 3000 V, gas temperature = 300 °C, gas flow rate = 3.0 L/min, acquisition range = 150–800 *m*/*z*. The initial elution profile of HPLC consisted of two solvents, 90% A (super distilled water with 0.5% formic acid) and 10% B (acetonitrile with 0.5% formic acid), isocratic elution 0–2 min, followed by a linear gradient to 45% B 2–17 min, at a flow rate at 0.3 mL/min. An Agilent Zorbax SB-C18 1.8 μm, 50 × 2.1 mm internal diameter column was used for separation.

### 4.4. Quantitative Determination

Dried leaves of *Z. marina* were cut into small pieces, homogenized and extracted with 50% aqueous methanol. The flavonoids from the extracts were analyzed using HPLC with DAD and LR- LCMS detection. For the quantitative analysis, four replicate samples of *Z. marina* were weighted (approx. 200 mg) and added to 15 mL sealed glass tubes. The leaves were extracted with 50% aqueous methanol (7 mL) at room temperature for 60 min. The extract was removed to another tube, and the process was repeated twice. To determine the volume of the combined extracts, the solution was transferred to a volumetric flask. Prior to the chromatographic analysis, the extracts were filtered through a Millipore membrane filter (0.45 μm). All the samples analyzed by HPLC were injected three times, and the results averaged.

Quantitative determination of the polyphenolic content in *Z. marina* was carried out using a calibration curve of luteolin (97.0% purity, Sigma-Aldrich, St. Louis, MO, USA). Six solutions with different concentrations were made to cover a broad concentration interval. The content of polyphenols is expressed in milligrams (mg) luteolin equivalents per gram dry weight (DW) of *Z. marina*. All chemicals used were of analytical grade. Acetonitrile (≥99.8%), methanol (≥99.9%), trifluoracetic acid (TFA) and luteolin reference standard were all purchased from Sigma-Aldrich (Sigma-Aldrich, St. Louis, MO, USA). Two-sided t-test assuming unequal variance with a *p*-value < 0.05 was used to determine if the means of different measurements were equal or not. Standard error bars were calculated using the STDV function in excel and represent one standard deviation (*n* = 4).

The HPLC method was validated by considering linearity, precision, limit of detection (LOD) and limit of quantification (LOQ). The results of the validation are presented in Table 5. LOD and LOQ were calculated by the standard deviation of the y-intercepts of the calibration curve (SD) and the slope (S) by using the equation LOD = 3.3 × SD/S and LOQ = 10 × SD/S. The square of the correlation coefficient (R^2^ = 0.9993–0.9995) showed good linearity.

### 4.5. Multivariate Analysis

Principal component analysis (PCA) is one of the most common techniques for the investigation of multivariate data [39]. PCA explores correlation patterns in multivariate data by linearly combining variables as principal components (PCs), each one successively explaining the variance maximally in the data with the constraint of being orthogonal to the others [39]. When the variables are strongly correlated, the first few PCs can describe the data without significant loss of information. Thus, the first principal component (PC1) is the linear combination of the original variables that explain most of the data matrix. PC2 is orthogonal to PC1 and contains most of the remaining variance [39].

The software Sirius (version 10) was used for multivariate analysis based on the qualitative and quantitative amounts of phenolics (**1**–**7**, **9**–**12**, RA and ZA) found in the collected *Z. marina* samples (Table 2, Figure 1) and the variables “depth” and “wave exposure”. The wave exposure values were log-transformed and all variables standardized to unit variance prior to PCA.

## 5. Concluding Remarks

The chemical ecology of seagrasses is complex, and a number of internal and external factors affect the phenolic chemistry besides the variables included in this study (latitude, depth, wave exposure and meadow position). We found that rosmarinic acid correlated moderately positively with depth, while the total flavonoids had an overall strong negative correlation with depth—possibly reflecting a lack of stress-induced conditions with increasing depth, rather than a different response to light conditions. At a molecular level, the flavonoids were separated into two groups; one group is well described by the variables depth and wave exposure, while the other group was not well described by these two variables. The latter could reflect biosynthetic dependencies within the flavonoids or responses to other unrevealed factors. We found a higher flavonoid/RA ratio in the periphery of the meadow—contrary to what was seen in the center of a meadow. This could be related to higher expected plant stress in the periphery of a meadow. Hence, the flavonoid/RA ratio within a seagrass meadow may be a simple biochemical marker or molecular index tool of seagrass health and warrants further exploration.

## Figures and Tables

**Table 1:** Flavonoids and phenolic acids in *Zostera marina* samples collected along a gradient of latitude.

Location ^1^	Compound ^2^												
	ZA	1	2	3	4	5	6	7	RA	9	10	11	12
Vestfold (A) (59° N 10° E)	+	+	+	+	+		+		+	+	+		+
Hordaland (B) (60° N 5° E)	+	+	+		+			+	+	+	+		+
Møre and Romsdal (C-L) (~63° N 7° E)	+	+	+	+	+		+	+	+	+	+		+
Finnmark (M, N) (~70° N 27° E)	*t*	+	+	+	+	+	+	*t*	+		+	+	*t*

*t* = traces < 0.01 mg/g, ^1^ For sample localities see Section 4.1. ^2^ Structures of individual flavonoids (**1**–**7**, **9**–**12**) and phenolic acids (ZA, RA) see Figure 1.

**Table 2:** Quantitative amounts (mg/g Lut. Eq.) of phenolics found in *Zostera marina* leaves sampled from different parts of Norway (see Section 4.1).

	ZA	1	2	3	4	5	6	7	RA	9	10	11	12	TF
A	0.36 ± 0.01	3.12 ± 0.10	0.41 ± 0.01	0.11 ± 0.03	0.74 ± 0.23		0.31 ± 0.04		0.83 ± 0.07	1.13 ± 0.12	3.92 ± 0.42		0.50 ± 0.03	10.23 ± 0.77
B	0.32 ± 0.06	3.36 ± 0.11	0.59 ± 0.02		0.44 ± 0.02			0.26 ± 0.02	0.51 ± 0.08	2.07 ± 0.18	2.02 ± 0.20		0.75 ± 0.04	9.49 ± 0.60
C_1_	0.05 ± 0.001	2.21 ± 0.29	0.18 ± 0.04		0.12 ± 0.04			<0.01	0.73 ± 0.12	0.76 ± 0.09	0.96 ± 0.22		0.33 ± 0.05	4.58 ± 0.69
C_2_	0.06 ± 0.02	3.05 ± 0.18	0.53 ± 0.02		0.58 ± 0.14		0.04 ± 0.01	0.05 ± 0.02	1.73 ± 0.13	1.49 ±0.17	2.56 ± 0.4		0.88 ± 0.08	9.18 ± 1.02
C_3_	0.03 ± 0.002	1.84 ± 0.14	0.19±0.01		0.10 ± 0.04			0.05 ± 0.02	0.81 ± 0.08	0.67 ± 0.13	0.55 ± 0.13		0.21 ± 0.04	3.61 ± 0.51
D_1_	0.12 ± 0.01	4.73 ± 0.22	0.18 ± 0.01		0.15 ± 0.04			0.06 ± 0.02	0.96 ± 0.14	0.49 ± 0.07	0.35 ± 0.05		0.06 ± 0.01	6.03 ± 0.39
D_2_	0.21 ± 0.01	5.45 ± 0.17	0.46 ± 0.02		0.74 ± 0.21			0.05 ± 0.02	0.50 ± 0.06	0.38 ± 0.07	1.01 ± 0.19		0.05 ± 0.02	8.14 ± 0.67
E_1_	0.05 ± 0.003	2.18 ± 0.11	0.21 ± 0.01		0.06 ± 0.05			<0.01	0.12 ± 0.07	1.16 ± 0.21	0.74 ± 0.21		0.32 ± 0.04	4.70 ± 0.59
E_2_	0.13 ± 0.03	2.97 ± 0.11	0.38 ± 0.02		0.07 ± 0.04			<0.01	0.31 ± 0.08	0.90 ± 0.14	0.78 ± 0.15		0.39 ± 0.04	5.51 ± 0.51
F_1_	0.16 ± 0.01	3.25 ± 0.10	0.23 ± 0.003		0.40 ± 0.09		0.05 ± 0.04	0.06 ± 0.02	1.24 ± 0.17	1.31 ± 0.13	0.84 ± 0.09		0.46 ± 0.03	6.61 ± 0.39
F_2_	0.08 ± 0.004	2.76 ± 0.16	0.18 ± 0.01		0.13 ± 0.05			0.03 ± 0.01	0.53 ± 0.06	1.00 ± 0.15	0.32 ± 0.08		0.23 ± 0.03	4.65 ± 0.49
G_1_	0.08 ± 0.01	3.51 ± 0.17	0.33 ± 0.01	0.09 ± 0.04	1.28 ± 0.15		0.17 ± 0.04	0.13 ± 0.003	1.41± 0.04	2.48 ± 0.07	1.63 ± 0.16		0.59 ± 0.05	10.20 ± 0.35
G_2_	0.38 ± 0.02	4.25 ± 0.08	0.18 ± 0.01		0.17 ± 0.01			0.04 ± 0.02	1.46 ± 0.18	0.14 ± 0.01	0.12 ± 0.02			4.90 ± 0.11
H	0.14 ± 0.02	4.60 ± 0.04	0.16 ± 0.003		0.11 ± 0.01			0.07 ± 0.01	1.80 ± 0.05	0.75 ± 0.02	0.16 ± 0.01		0.16 ± 0.01	5.83 ± 0.07
I	0.21 ± 0.01	4.71 ± 0.07	0.27 ± 0.003		0.36 ± 0.02			0.16 ± 0.02	4.53 ± 0.31	0.12 ± 0.005	0.22 ± 0.01			5.84 ± 0.05
J_1_	0.20 ± 0.02	2.42 ± 0.21	0.23 ± 0.04		0.47 ± 0.11			0.08 ± 0.02	1.20 ± 0.06	1.36 ± 0.17	1.03 ± 0.17		0.47 ± 0.06	6.08 ± 0.74
J_2_	0.18 ± 0.01	3.78 ± 0.38	0.25 ± 0.01		0.19 ± 0.06			0.07 ± 0.02	0.73 ± 0.05	1.43 ± 0.22	0.40 ± 0.07		0.30 ± 0.03	6.42 ± 0.77
K_1_	0.05 ± 0.002	2.87 ± 0.11	0.30 ± 0.01		0.11 ± 0.04			0.04 ± 0.02	0.47 ± 0.02	0.94 ± 0.13	0.25 ± 0.05		0.19 ± 0.02	4.69 ± 0.38
K_2_	0.06 ± 0.005	3.08 ± 0.24	0.54 ± 0.06		0.17 ± 0.06			0.03 ± 0.02	0.79 ± 0.20	0.92 ± 0.18	0.47 ± 0.11		0.31 ± 0.05	5.52 ± 0.70
K_3_	0.01 ± 0.002	4.87 ± 0.12	0.57 ± 0.02		0.33 ± 0.08			0.09 ± 0.03	0.32 ± 0.10	0.10 ± 0.02	0.87 ± 0.11			6.84 ± 0.31
K_4_	0.09 ± 0.002	3.13 ± 0.19	0.39 ± 0.01		0.25 ± 0.06		0.08 ± 0.02	0.11 ± 0.02	1.44 ± 0.11	1.14 ± 0.16	0.62 ± 0.09		0.34 ± 0.03	6.05 ± 0.56
L	0.15 ± 0.01	3.40 ± 0.19	0.16 ± 0.01		0.13 ± 0.04			0.07 ± 0.03	0.91 ± 0.01	0.50 ± 0.08	0.41 ± 0.07		0.09 ± 0.02	4.76 ± 0.42
M	<0.01	0.84 ± 0.14	0.11 ± 0.04		<0.01			<0.01	<0.01		1.40 ± 0.24			2.35 ± 0.41
N	<0.01	1.83 ± 0.09	0.06 ± 0.005	0.24 ± 0.02	1.37 ± 0.20	0.06 ± 0.01	0.31 ± 0.06	<0.01	0.86 ± 0.14		3.58 ± 0.35	0.11 ± 0.01	<0.01	7.57 ± 0.58

The quantitative amouns (mg/g Lut. Eq.) are presented as mean ± standard deviation of four replicated HPLC analysis (*n* = 4), see Section 4.4.

**Table 3:** Principal component analysis (PCA) partial correlation coefficient table for the variables; depth, wave exposure, rosmarinic acid (RA) and zosteric acid (ZA)—describing 71% of the variance.

Variables	Depth	Wave Exposure	Rosmarinic Acid (RA)	Zosteric Acid (ZA)
Depth	1	−0.11	0.52	−0.47
Wave exposure		1	0.79	0.93
Rosmarinic acid (RA)			1	0.51
Zosteric acid (ZA)				1

Correlation coefficient category: strong (>0.75), moderate (0.75–0.5), weak (<0.5) [40].

**Table 4:** Overview of locations, depths and wave exposure (value and classes) for the collected *Zostera marina* sampling (see Figure 8).

	Collection Sites	Region, Municipality	Latitude	Longitude	Depth (cm)	Wave Exposure (m^2^/s)	Wave Exposure Class	Meadow Position
A	Ølbergholmen south	Vestfold, Larvik	59.00681	10.13163	50	34,131	Sheltered	P
B	Espegrend	Hordaland, Bergen	60.16120	5.13203	40–100	3739	Extremely sheltered	P
C_1_	Inner Sykkylvsfjorden	Møre and Romsdal, Sykkylven	62.34095	6.58465	580	3334	Extremely sheltered	C
C_2_			62.34150	6.58665	90	3357	Extremely sheltered	P
C_3_			62.34428	6.58472	350	3333	Extremely sheltered	P
D_1_	Volsund	Møre and Romsdal, Herøy	62.36319	5.66683	280	9561	Very sheltered	C
D_2_			62.36387	5.66637	160	6332	Very sheltered	P
E_1_	Hjartevika, Sykkylvsfjorden	Møre and Romsdal, Sykkylven	62.37240	6.57199	300	4253	Very sheltered	C
E_2_			62.37267	6.57237	250	4120	Very sheltered	C
F_1_	Bøleira, Innfjorden	Møre and Romsdal, Vestnes	62.49767	7.55012	400	6003	Very sheltered	*n.r.*
F_2_			62.49796	7.54864	250	6237	Very sheltered	*n.r.*
G_1_	Stormalen	Møre and Romsdal, Sandøy	62.75238	6.48934	570	72,851	Sheltered	C
G_2_			62.75089	6.48848	390	63,739	Sheltered	C
H	Storholmen	Møre and Romsdal, Sandøy	62.75439	6.40991	540	21,214	Sheltered	C
I	Malesanden	Møre and Romsdal, Sandøy	62.77301	6.47301	230	33,657	Sheltered	C
J_1_	Fannefjorden	Møre and Romsdal, Molde	62.78916	7.71018	360	4050	Very sheltered	*n.r.*
J_2_			62.78957	7.70725	140	4050	Very sheltered	*n.r.*
K_1_	Between Nautneset and Halsan (Gossa)	Møre and Romsdal, Aukra	62.81113	6.91111	450	2690	Extremely sheltered	C
K_2_			62.81136	6.91224	640	3177	Extremely sheltered	C
K_3_			62.81137	6.91039	280	2788	Extremely sheltered	P
K_4_			62.81167	6.91193	740	3257	Extremely sheltered	P
L	Kalsvika, Bud	Møre and Romsdal, Sandøy	62.90180	6.92823	320	51,652	Sheltered	*n.r.*
M	Munkefjorden	Finnmark, South-Varanger,	69.66068	29.51747	120–150	7449	Very sheltered	P
N	Rafsbotn	Finnmark, Alta	70.01454	23.48984	50	25,053	Sheltered	P

Wave exposure values were extracted from a model developed by NIVA in the National Program for Mapping of Biodiversity—Coast. Meadow positions are recorded as either in the center (C, i.e., in the middle) or periphery (P, i.e., towards the border) of the meadow [52], *n.r.* = not registered.

**Table 5:** Luteolin calibration curves, test range, limit of detection (LOD) and limit of quantification (LOQ) for HPLC analysis of *Zostera marina*.

Standard	Calibration Curve ^a^	Test Range ^b^	R^2^	LOD ^b^	LOQ ^b^
Luteolin 1	Y = 27353x + 57.68	0.0048–0.367	0.9993	0.013	0.038
Luteolin 2	Y = 29977x + 63.33	0.0061–0.4891	0.9995	0.013	0.041

^a^ Y = peak area, x = concentration (mM) ^b^ (mM).

## Data Availability

Raw data aviable at: Dataverse NO, UiB Open Research Data: Dybsland, Cecilie Sævdal; Enerstvedt, Kjersti Hasle; Jordheim, Monica, 2021, “Replication Data for: Variation in phenolic chemistry in Zostera marina seagrass along environmental gradients”, https://doi.org/10.18710/4UCFSF.
